# Effects of Dietary β-Mannanase Supplementation on Growth Performance, Lipid Fraction Contents, and Physiological Responses in Broiler Chicks

**DOI:** 10.3390/biology15110821

**Published:** 2026-05-23

**Authors:** Jung-Min Park, Byoung-Ki An, Seok-Hyeon Cho, Chang-Won Kang

**Affiliations:** 1 Department of Food Marketing and Safety, Konkuk University, Seoul 05029, Republic of Korea; pjm0321@konkuk.ac.kr; 2 Animal Resources Research Center, Konkuk University, Seoul 05029, Republic of Korea; zdream75@naver.com (S.-H.C.); kkucwkang@konkuk.ac.kr (C.-W.K.)

**Keywords:** β-mannanase, broiler chicks, growth performance, hepatic lipid fractions, physiological responses

## Abstract

This experiment was conducted to evaluate the dietary effects of β-mannanase on growth performance, cecal ammonia concentration, lipid fraction contents and physiological responses in broiler chicks. A total of nine hundred 1 d old Ross male broiler chicks were assigned to six groups arranged in a 3 × 2 factorial of three levels of energy and CP [standard energy + standard CP (HEHP), 75 kcal/kg diet reduction in energy + standard CP (REHP), 75 kcal/kg diet reduction in energy + 1% reduction in CP (RERP)], with or without β-mannanase (0 or 0.05%). As dietary energy and CP decreased, growth performance also decreased. The growth and feed conversion ratio in chicks fed diets containing β-mannanase significantly improved. No significant differences were observed in the levels of serum enzymes and the relative weight and length of each fraction of small intestine among groups, except for the liver. The concentration of cecal ammonia in chicks fed diets containing β-mannanase was significantly lower than those of corresponding groups without β-mannanase. The levels of serum and hepatic lipid fractions were not influenced by supplementing β-mannanase to the diets. These results indicated that dietary β-mannanase may improve growth performance and utilization of dietary energy and protein in broiler chicks.

## Simple Summary

Reducing dietary nutrient levels is commonly used to lower feed costs in poultry production, but it often results in reduced growth performance. This study evaluated whether β-mannanase supplementation could alleviate these effects in broiler chicks. The results showed that lowering nutrient levels impaired growth performance, whereas β-mannanase improved body weight gain and feed efficiency. In addition, the enzyme reduced cecal ammonia concentration, indicating improved nutrient utilization, without adverse effects on bird health. These findings suggest that β-mannanase supplementation may help maintain productivity in broiler chicks fed reduced-nutrient diets, offering a practical approach for cost-effective and sustainable poultry production.

## 1. Introduction

Exogenous enzymes act to break down fibrous, anti-nutritive carbohydrates such as non-starch polysaccharides (NSPs) in poultry diets [1]. It has been well known that NSP enzymes reduce digesta viscosity, increase nutrient digestibility and improve gut health [2]. β-mannans, one of the NSPs, are mainly found in the hull and fiber fractions of soybean meal (SBM), sesame meal and guar meal [3]. Some studies have demonstrated adverse effects of dietary β-mannan found in guar gum and SBM [4,5]. The galactomannan in diet increases digesta viscosity [6], which may limit the access of digestive enzymes to their substrates and therefore decreases nutrients digestibility [7].

Dietary β-mannanase enzyme is widely utilized in poultry diets to alleviate the anti-nutritional effects of β-mannans [8,9]. Latham et al. [10], studying the efficacy of β-mannanase in broiler diets, found that the addition of this enzyme in diets with reduced ME and CP promoted similar growth performance to those fed a diet with adequate energy and nutrient levels. β-mannanase supplementation have been shown to increase AMEn and have beneficial effects on growth performance when added into poultry diets [11,12]. In a previous study, we reported that egg production of hens fed reduced ME and CP diets were similar compared to an energy- and CP-sufficient diet when β-mannanase was added into the diet [13]. Mechanistically, high digesta viscosity induced by β-mannans can physically interfere with the emulsification of dietary fats and the formation of mixed micelles, thereby reducing lipid absorption efficiency. While β-mannans have been reported to affect fat digestibility and lower total blood cholesterol levels [14], the dietary effects of β-mannanase supplementation on blood lipid profiles remain inconsistent [15,16]. Only limited information is available on the effects of β-mannanase supplementation on lipid metabolism in avian species. Thus, the present study was intended to validate whether there is an interaction between dietary nutrition density and β-mannanase supplementation on lipid fraction contents and physiological responses in broiler chicks.

## 2. Materials and Methods

All animal care procedures were approved by the Institutional Animal Care and Use Committee at Konkuk University (protocol code: KU13188, date of approval: 3 April 2020).

### 2.1. Animal Husbandry, Study Design and Diets

On the day of hatch, 308 Ross male broiler chicks were received from a local hatchery (Yangji Hatchery, Pyeongtaek, Republic of Korea). They were weighed individually and randomly assigned into 30 pens with 30 chicks per pen (1.8 m × 1.8 m) and stocking density was set at 0.11 m^2^. In total, nine hundred chicks were housed on rice husks as a bedding material with 23/1 light/dark cycle of 20 lx intensity throughout the experimental period and fed one of six diets for 35 d of the rearing period. Five replicates of 30 chicks were randomly assigned to six dietary treatments based on a 3 × 2 factorial arrangement. Diet and water were provided ad libitum. The temperature of the facility was maintained at 33 °C during the first week posthatch and gradually decreased to reach 25 °C at 3 weeks and kept thereafter.

β-mannanase (CTCZYME^®^, 800,000 U/kg) was provided by CTCBIO Inc. (Seoul, Republic of Korea). The present experiment consisted of six dietary treatments which were given for 35 days (starter 1 to 21 days, finisher 22 to 35 days). The three starter diets containing different levels of AMEn and CP were made as follows: HEHP, a corn–soybean meal-based diet that met or exceeded NRC [17] nutrient requirements containing 3075 kcal/kg of AMEn and 21.5% CP; REHP, a diet that contained lower energy than HEHP with 3000 kcal/kg of AMEn and 21.5% CP; and RERP, a diet that contained lower energy and CP levels than HEHP with 3000 kcal/kg of AMEn and 20.4% CP (Table 1).

The three finisher diets containing different levels of AMEn and CP were also made as follows: HEHP, a diet containing 3120 kcal/kg of AMEn and 20.0% CP; REHP, a diet that containing lower energy than HEHP with 3050 kcal/kg of AMEn and 20.0% CP; and RERP, a diet that containing lower energy and CP levels than HEHP with 3050 kcal of AMEn and 19.0% CP per kg feed. Each of the experimental diets was supplemented with either 0 or 0.05% β-mannanase (Table 2).

### 2.2. Sample Collection and Analysis

The chicks and feed were weighed weekly per pen to calculate live body weight (BW), weight gain, feed intake and feed conversion ratio (FCR) on a pen basis. At the end of the experimental period, ten chicks with similar BW per treatment were selected and weighed individually. The blood was drawn from wing veins using sterilized syringes for determination of the blood profiles. The levels of serum albumin, glutamic oxaloacetic transaminase (GOT), and glutamic pyruvic transaminase (GPT) were measured according to the colorimetric methods using a biochemical analyzer (Hitachi modular system, Hitachi Ltd., Tokyo, Japan).

At necropsy, liver, spleen, bursa of Fabricius, abdominal fat, and right breast muscle were immediately removed and weighed. The relative weights of these organs were expressed as a percentage of live BW. Then, liver and serum were frozen for subsequent determination of the contents of each lipid fraction. In addition, the fractions of duodenum, jejunum and ileum were also sampled, weighed and measured in length.

The digesta of duodenum, jejunum and ileum were collected to measure viscosity. Digesta were centrifuged at 9000 rpm for 10 min at 4 °C and 0.5 mL of supernatant was placed in the viscometer (LVDL-II+P CP, Brookfield, New York, NY, USA). The viscometer was set at 10 rpm for evaluation, and the viscosity was recorded after 30 s. Ammonia concentration of the cecal digesta was measured by following the procedure described in the ammonia assay kit (Product code AA0100, Sigma-Aldrich, Saint Louis, MO, USA). About 0.1 mL diluted cecal digesta sample was placed into a cuvette and mixed with 1 mL ammonia assay reagent and then incubated for 5 min at 35 °C. After that, absorbance was read at 340 nm against blank samples. And then, 0.01 mL of L-glutamate dehydrogenase solution was added to each cuvette and incubated at 35 °C for 5 min. The absorbance of each solution was measured again at 340 nm.

The contents of each lipid fraction from liver and serum were measured following a previously described method by Park et al. [18] with some modifications. The total lipids were extracted from liver and serum using chloroform/methanol (2:1, *v*/*v*) as described by Folch et al. [19]. The contents of each lipid fraction in liver and serum were separated by thin-layer chromatography on silica gel chromarods using hexane:diethylether:formic aid (85:15:0.5, *v*/*v*) as developing solvents and quantified by IATRO SCAN (TH-10 TLC/FID analyzer, Iatron laboratory Inc., Tokyo, Japan).

### 2.3. Statistical Analysis

Data were analyzed as a complete randomized design by two-way ANOVA using the GLM procedure of SAS software (version 9.4; SAS Institute Inc., Cary, NC, USA) [20] to determine the effects of dietary nutrients levels on enzymes and their interactions. Pen was considered as an experimental unit for growth performance. The individual bird was considered experimental unit for the rest measurements A statistical significance was preset at *p* < 0.05 unless otherwise stated. When significant interactions occurred, means among treatments were compared in a pairwise manner using the probability of differences (PDIFF) option of SAS.

## 3. Results

The effects of dietary nutrients levels and β-mannanase supplementation on growth performance of broiler chicks are presented in Table 3. No interaction between dietary nutrient level and β-mannanase supplementation was observed for final BW (*p* = 0.19). However, final BW was significantly affected by both dietary nutrient level (*p* < 0.01) and β-mannanase supplementation (*p* < 0.01), with higher values observed in the high-nutrient group and in birds supplemented with β-mannanase. No interaction between dietary nutrient level and β-mannanase supplementation was observed for BW gain during any of the growth periods (*p* > 0.05). Similar to final BW, BW gain was significantly influenced by both dietary nutrient level (*p* < 0.01) and β-mannanase supplementation (*p* < 0.01). Specifically, β-mannanase supplementation significantly increased BW gain during the starter (*p* < 0.05), finisher (*p* < 0.01), and overall periods (*p* < 0.01). No interactions between β-mannanase and nutrients levels were observed for feed intake during the total rearing period. The interaction between β-mannanase and nutrients levels was found for FCR during the finisher and total rearing period (*p* < 0.01). β-mannanase and nutrients effects were shown in the FCR during the total rearing period, respectively (*p* < 0.01; *p* < 0.01).

There were no interaction effects between β-mannanase and nutrients levels on the relative weights of various organs including liver, spleen, bursa of Fabricius, abdominal fat, breast muscle and legs. The dietary nutrients levels and β-mannanase supplementation had no effects on the relative weights and lengths of intestinal fractions. No differences or interactions were observed for serum albumin, GOT and GPT.

The interaction between β-mannanase and nutrients levels was not observed for digesta viscosity of duodenum, jejunum and ileum. The ileal digesta viscosity was significantly influenced by dietary nutrients levels (*p* < 0.01). There were trends for decreased digesta viscosity of jejunum and ileum with β-mannanase supplementation, respectively (*p* = 0.07; *p* = 0.09). No interactions between β-mannanase and nutrients levels were observed for ammonia concentration; however, there was a decrease (*p* < 0.01) in cecal ammonia concentration with β-mannanase supplementation (Table 4).

The effects of dietary nutrients levels and β-mannanase supplementation on contents of lipid fractions in serum and liver are presented in Table 5. No interactions between β-mannanase and nutrients levels were observed for contents of cholesterol, triacylglycerol and phospholipid. Dietary nutrients levels and β-mannanase supplementation did not influence the contents of lipid fractions in serum and liver.

## 4. Discussion

It is well known that commercial enzyme products play important roles in reducing anti-nutritional factors that exist in plant origin feedstuffs such as cell wall components and NSP [2]. The increased performance of poultry supplemented with enzyme products has been linked to an improvement in digestibility of energy and nutrients. β-mannanase has also been shown to improve feed efficiency of broiler chicks and laying hens [21]. The addition of β-mannanase has been shown to lead to the improvement in the digestibility of nutrients like CP [22], amino acids [23], and phosphorus [24].

Enhanced feed utilization can reduce levels of some nutrients and energy in broiler diets without losses in growth performance. In addition, with the release of energy, the inclusion of energetic feedstuffs can be decreased, which will be reflected in reduction in feed costs. Several studies investigating the effects of β-mannanase on energy utilization showed it can increase energy values of the diets when fed to broiler chicks [11,25] and laying hens [16]. The release of the mannose in the small intestine by the addition of β-mannanase would lead to the increase in the ME of the diet. Low-energy diets supplemented with β-mannanase revealed similar or superior performance to high-energy diets without β-mannanase in broiler chicks. When broiler chicks were fed diets containing 97 kcal lower energy levels than a control diet, with the supplementation of β-mannanase, the growth performance was similar to those fed a control diet [10]. β-mannanase (endo-1,4-β-mannanase) used in this study cleaves randomly within the β-D-1,4 mannopyranoside linkages, and is speculated to produce mannan-oligosaccharides and a small amount of mannose when added into diets [26]. The results of the present study demonstrate the beneficial effects of the use of β-mannanase on growth parameters in broiler chicks. The BW gain and FCR of chicks fed REHP or RERP diets supplemented with β-mannanase were similar to those of chicks fed HEHP diets without β-mannanase. It can be speculated that the improved energy utilization as a result of β-mannanase supplementation might have partially improved the growth performance [27,28].

Dietary nutrients levels and β-mannanase supplementation had no effects on the relative weights of various organs and intestinal fractions. Previous results on the dietary effects of β-mannanase on organ weights have been contradictory. Li et al. [22] reported that β-mannanase supplementation decreased the relative weight of liver and intestine in broiler chicks. On the contrary, Mohammadigheisar et al. [29] found that adding β-mannanase to broiler diets did not affect the relative weights of various organs and blood profiles. No differences or interactions were observed for serum albumin, GOT and GPT in the present study. GOT and GPT are hepatic enzymes measured in blood tests to assess liver health and tissue damage and are a valuable tool to determine safe applications for new feedstuffs and feed additives. In any event, dietary β-mannanase did not affect blood profiles, indicating its safe use for broiler chicks as confirmed in several previous studies.

The use of feed ingredients with high amounts of β-mannan may increase the digesta viscosity in poultry [21]. β-mannan binds a large quantity of water leading to increased viscosity and reduction in endogenous enzyme–substrate interactions. The reduction in digesta viscosity is one benefit of β-mannanase supplementation, with different enzymes working on the soluble polysaccharide content, thereby reducing viscosity and improving nutrient absorption and utilization. Latham et al. [10] reported that β-mannanase added in broiler diets with varying galactomannan levels improved growth performance and reduced digesta viscosity. β-mannanase supplementation in Pekin duck diets with guar resulted in a reduction in digesta viscosity, but, in diets without guar, the same enzyme did not significantly impact viscosity in the intestinal segment [30]. In present study, there were trends for decreased digesta viscosity of the jejunum and ileum with β-mannanase supplementation without a significant difference. This discrepancy may be attributed to differences in animals, activity of β-mannanase and composition of experimental diets, particularly variations in mannan content. Cecal ammonia concentration decreased with β-mannanase supplementation in the present study. β-mannanase supplementation reduced excreted nitrogen and increased amino acid digestibility when fed to broiler chickens [23]. The reduction in ammonia concentration shown in present study might be related to improvement in CP digestibility. In a previous study using laying hens, there was a decrease in ammonia concentration with β-mannanase supplementation [13].

It has also been suggested that dietary β-mannanase may affect blood lipid profiles in poultry and pigs. Kim et al. [16] reported that β-mannanase supplementation into low- or high-mannan diets had no effects on the levels of total blood cholesterol and triacylglycerol in growing pigs. In contrast, Jang et al. [15] found that total cholesterol concentration significantly increased in weaning pigs fed the diet with β-mannanase. The concentrations of high-density lipoprotein cholesterol and low-density lipoprotein cholesterol also linearly increased with higher levels of β-mannanase. It has been referred to increased cholesterol synthesis, decreased cholesterol excretion, or both as possible mechanisms with respect to increases in blood cholesterol levels. In present study, neither dietary nutrients levels or β-mannanase supplementation influenced the contents of lipid fractions in serum and liver. Phospholipid and cholesterol are major constituents of hepatic cell membranes, facilitating cell function and vitality and modulating fluidity and structure [31]. Recently, Kim et al. [32] reported that β-mannanase supplementation in a high-mannan diet had no positive effect on fatty liver incidence in laying hens. Because there is a notable shortage of studies on lipid transport and metabolism, further study is required to clarify the dietary effect of this enzyme on lipid synthesis, degradation and redistribution into various organs.

## 5. Conclusions

Overall, when β-mannanase supplementation was incorporated in a low-energy and low-protein diet, broiler chicks were able to produce similar production performance. Dietary β-mannanase can decrease cecal ammonia levels, but neither dietary nutrient levels or β-mannanase supplementation influenced the contents of lipid fractions in serum and liver.

## Figures and Tables

**Table 1:** Formula and chemical composition of experimental diet (starter).

Ingredients (%)	HEHP	REHP	RERP
Yellow corn	55.83	57.83	59.88
Soybean meal (44%)	33.66	33.56	32.4
Corn gluten meal	2.96	2.81	1.59
Wheat bran	-	-	0.44
Tallow	3.88	2.12	2.00
Vit. + Min. mixture	0.20	0.20	0.20
Dicalcium phosphate	1.90	1.90	1.90
DL-methionine (98%)	0.17	0.17	0.17
Limestone	1.01	1.02	1.03
Choline chloride (50%)	0.09	0.09	0.09
Salt	0.30	0.30	0.30
Total	100	100	100
Calculated values			
TMEn, kcal/kg	3075	3000	3000
Crude protein, %	21.50	21.50	20.40
Ca, %	1.00	1.00	1.00
Available P, %	0.45	0.45	0.45
Lysine, %	1.14	1.14	1.10
Met + Cys, %	0.90	0.90	0.86

HEHP, a diet containing 3075 kcal/kg of AMEn and 21.5% CP; REHP, a diet containing 3000 kcal/kg of AMEn and 21.5% CP; RERP, a diet containing 3000 kcal/kg of AMEn and 20.4 CP; vitamin mixture provided the following nutrients per kg: vitamin A, 40,000,000 IU; vitamin D3, 8,000,000 IU; vitamin E, 10,000 IU; vitamin K3, 4000 mg; vitamin B1, 4000 mg; vitamin B2, 12,000 mg; vitamin B6, 6000 mg; vitamin B12, 20,000 µg; pantothenic acid, 20,000 mg; folic acid, 2000 mg; nicotinic acid, 60,000 mg; mineral mixture provided the following nutrients per kg: Fe, 30,000 mg; Zn, 25,000 mg; Mn, 20,000 mg; Co, 150 mg; Cu, 5000 mg; Ca, 250 mg; Se, 100 mg.

**Table 2:** Formula and chemical composition of experimental diet (finisher).

Ingredients (%)	HEHP	REHP	RERP
Yellow corn	60.22	62.03	65.33
Soybean meal (44%)	30.33	30.57	28.35
Corn gluten meal	2.67	2.29	1.77
Tallow	3.66	2.00	1.40
Vit. + Min. mixture	0.20	0.20	0.20
Lysine (98.5%)	-	-	-
Dicalcium phosphate	1.38	1.38	1.39
DL-methionine (98%)	0.06	0.06	0.06
Limestone	1.11	1.11	1.12
Choline chloride (50%)	0.07	0.07	0.07
Salt	0.30	0.30	0.30
Total	100.00	100.00	100.00
Calculated values			
TMEn, kcal/kg	3120	3050	3050
Crude protein, %	20.00	20.00	19.00
Ca, %	0.90	0.90	0.90
Available P, %	0.35	0.35	0.35
Lysine, %	1.10	1.10	0.99
Met + Cys, %	0.75	0.75	0.72

HEHP, a diet containing 3120 kcal/kg of AMEn and 20.0% CP; REHP, a diet containing 3050 kcal/kg of AMEn and 20% CP; RERP, a diet containing 3050 kcal/kg of AMEn and 19.0% CP; vitamin mixture provided the following nutrients per kg: vitamin A, 40,000,000 IU; vitamin D3, 8,000,000 IU; vitamin E, 10,000 IU; vitamin K3, 4000 mg; vitamin B1, 4000 mg; vitamin B2, 12,000 mg; vitamin B6, 6000 mg; vitamin B12, 20,000 µg; pantothenic acid, 20,000 mg; folic acid, 2000 mg; nicotinic acid, 60,000 mg; mineral mixture provided the following nutrients per kg: Fe, 30,000 mg; Zn, 25,000 mg; Mn, 20,000 mg; Co, 150 mg; Cu, 5000 mg; Ca, 250 mg; Se, 100 mg.

**Table 3:** Effects of dietary β-mannanase on growth performance in broiler chicks.

Diet											
	Initial BW, g/bird	Final BW, g/bird	BW Gain, g/bird/d			Feed Intake, g/bird/d			FCR		
			1–21 d	22–35 d	1–35 d	1–21 d	22–35 d	1–35 d	1–21 d	22–35 d	1–35 d
HEHP	45.60	1859.10	31.02 ^a^	82.90 ^a^	58.50 ^a^	53.78	146.26	86.38	1.73	1.76 ^b^	1.75 ^c^
REHP	45.37	1788.15	29.69 ^ab^	80.13 ^a^	56.23 ^ab^	53.62	146.39	88.08	1.80	1.83 ^b^	1.83 ^b^
RERP	45.28	1701.37	28.68 ^b^	75.27 ^b^	53.41 ^b^	53.69	145.35	86.13	1.88	1.93 ^a^	1.92 ^a^
SEM	0.39	19.92	0.37	1.03	0.66	0.62	1314	1.05	0.03	0.02	0.02
*p* value	0.83	<0.01	<0.01	<0.01	<0.01	0.98	0.74	0.39	0.02	<0.01	<0.01
Enzyme											
0	45.66	1745.61	29.34	77.56	54.83	53.13	146.28	85.73	1.81	1.89	1.87
0.05	45.16	1820.13	30.35	81.31	57.26	54.26	145.25	88.00	1.79	1.79	1.79
SEM	0.32	16.27	0.31	0.84	0.57	0.52	0.93	0.90	0.03	0.01	0.01
*p* value	0.25	<0.01	0.02	<0.01	<0.01	0.12	0.37	0.09	0.42	<0.01	<0.01
Diet × Enzyme											
HEHP + 0	46.02	1791.11	30.35	79.13	56.29	52.80	148.46 ^a^	85.34	1.74	1.88 ^ab^	1.84 ^ab^
HEHP + 0.05	45.19	1927.09	31.70	86.68	60.71	54.76	142.06 ^b^	87.42	1.73	1.64 ^c^	1.67 ^c^
REHP+ 0	45.94	1762.09	29.24	78.95	55.37	53.77	146.38 ^ab^	86.66	1.84	1.86 ^ab^	1.85 ^ab^
REHP+ 0.05	44.79	1814.21	30.13	81.31	57.09	53.46	146.40 ^ab^	89.50	1.78	1.80 ^b^	1.80 ^b^
RERP + 0	45.04	1683.65	28.12	74.61	52.84	52.83	144.00 ^ab^	85.20	1.88	1.93 ^a^	1.91 ^a^
RERP + 0.05	45.51	1719.10	29.23	75.93	53.99	54.55	146.70 ^ab^	87.07	1.87	1.93 ^a^	1.92 ^a^
SEM	0.52	28.18	0.50	1.46	0.94	0.92	1.62	1.48	0.05	0.03	0.02
*p* value	0.30	0.19	0.91	0.10	0.22	0.35	0.03	0.95	0.82	<0.01	<0.01

HEHP, a starter diet containing 3075 kcal/kg of AMEn and 21.5% CP, a finisher diet contained 3120 kcal/kg of AMEn and 20.0% CP; REHP, a starter diet containing 3000 kcal/kg of AMEn and 21.5% CP, a finisher diet contained 3050 kcal/kg of AMEn and 20% CP; RERP, a starter diet containing 3000 kcal/kg of AMEn and 20.4 CP, a finisher diet contained 3050 kcal/kg of AMEn and 19.0% CP; SEM, standard error of measurement; ^a–c^ mean values with different superscripts within a column differ significantly (*n* = 5).

**Table 4:** Effects of dietary β-mannanase on intestinal ammonia concentration and digesta viscosity in broiler chicks.

Diet	Ammonia Concentration, µg/mL	Viscosity, mPas		
		Duodenum	Jejunum	Ileum
HEHP	1.52	1.27	2.77	3.47 ^a^
REHP	1.47	2.12	2.54	2.56 ^b^
RERP	1.53	2.46	2.47	2.32 ^b^
SEM	0.04	0.17	0.15	0.14
*p* value	0.50	0.34	0.36	>0.01
Enzyme				
0	1.59	2.37	2.76	2.92
0.05	1.42	2.13	2.42	2.64
SEM	0.03	0.14	0.12	0.11
*p* value	<0.01	0.24	0.07	0.09
Diet × Enzyme				
HEHP + 0	1.68	2.37	2.94	3.71
HEHP + 0.05	1.35	1.96	2.59	3.22
REHP+ 0	1.51	2.02	2.66	2.72
REHP+ 0.05	1.43	2.03	2.42	2.40
RERP + 0	1.59	2.52	2.67	2.33
RERP + 0.05	1.48	2.39	2.27	2.30
SEM	0.06	0.24	0.21	0.20
*p* value	0.09	0.82	0.93	0.50

HEHP, a starter diet containing 3075 kcal/kg of AMEn and 21.5% CP, a finisher diet contained 3120 kcal/kg of AMEn and 20.0% CP; REHP, a starter diet containing 3000 kcal/kg of AMEn and 21.5% CP, a finisher diet contained 3050 kcal/kg of AMEn and 20% CP; RERP, a starter diet containing 3000 kcal/kg of AMEn and 20.4 CP, a finisher diet contained 3050 kcal/kg of AMEn and 19.0% CP; SEM, standard error of measurement; ^a–b^ mean values with different superscripts within a column differ significantly (*n* = 5).

**Table 5:** Effects of dietary β-mannanase on liver and serum lipid fractions of broiler chicks.

Diet	Liver, mg/g			Serum, mg/dL		
	Cholesterol	Triacylglycerol	Phospholipid	Cholesterol	Triacylglycerol	Phospholipid
HEHP	5.40	3.42	21.69	36.84	122.04	460.04
REHP	5.41	3.05	21.71	38.20	122.41	448.89
RERP	5.49	3.27	20.62	37.36	123.24	447.61
SEM	0.06	0.12	0.53	0.85	5.92	23.07
*p* value	0.51	0.12	0.27	0.53	0.98	0.92
Enzyme						
0	5.44	3.28	21.53	37.92	126.45	451.95
0.05	5.43	3.21	21.15	37.01	120.00	452.41
SEM	0.05	0.10	0.43	0.69	4.83	18.84
*p* value	0.82	0.63	0.54	0.37	0.35	0.99
Diet × Enzyme						
HEHP + 0	5.41	3.41	22.06	36.51	127.44	463.74
HEHP + 0.05	5.40	3.42	21.31	37.16	120.64	456.34
REHP+ 0	5.43	3.14	22.41	38.74	126.47	448.71
REHP+ 0.05	5.39	2.95	21.01	37.60	118.34	449.06
RERP + 0	5.49	3.29	20.10	38.50	125.46	443.39
RERP + 0.05	5.49	3.26	21.14	36.22	121.02	451.83
SEM	0.09	0.17	0.74	1.20	8.37	32.34
*p* value	0.97	0.94	0.25	0.48	0.97	0.97

HEHP, a starter diet containing 3075 kcal/kg of AMEn and 21.5% CP, a finisher diet contained 3120 kcal/kg of AMEn and 20.0% CP; REHP, a starter diet containing 3000 kcal/kg of AMEn and 21.5% CP, a finisher diet contained 3050 kcal/kg of AMEn and 20% CP; RERP, a starter diet containing 3000 kcal/kg of AMEn and 20.4 CP, a finisher diet contained 3050 kcal/kg of AMEn and 19.0% CP; abbreviations: SEM, standard error of measurement; values are presented as mean (*n* = 5).

## Data Availability

The original contributions presented in the study are included in the article; further inquiries can be directed to the corresponding author.
